# Case Report: Upadacitinib for Acrodermatitis continua of Hallopeau: a case series with literature review

**DOI:** 10.3389/fimmu.2026.1903377

**Published:** 2026-10-14

**Authors:** Yu-ting Gan, Shi Yan, Wei-na Xie, Yi Liu, Feng-lin Chen

**Affiliations:** 1 Department of Dermatology, The First Affiliated Hospital of Chongqing College of Traditional Chinese Medicine, Chongqing, China; 2 Chongqing University of Chinese Medicine, Chongqing, China

**Keywords:** Acrodermatitis continua of Hallopeau, case series, JAK inhibitor, literature review, tofacitinib, upadacitinib

## Abstract

**Background:**

Acrodermatitis continua of Hallopeau (ACH) is a relatively rare chronic skin disorder often associated with immune system abnormalities. Conventional systemic therapies and biologics have limited efficacy and are prone to relapse, highlighting the urgent need for new treatment options. Upadacitinib, a highly selective JAK1 inhibitor, has shown promising results in various inflammatory skin diseases; however, its application in ACH has rarely been reported.

**Methods:**

A retrospective analysis was conducted on 6 ACH patients treated with upadacitinib at our outpatient clinic from 2023 to 2025. Clinical data, including demographics, disease duration, sites involved, prior treatments, dosage, treatment duration, DLQI scores, and adverse events, were collected. Meanwhile, a narrative literature search was performed in PubMed, Web of Science, and Embase to identify studies on JAK inhibitors in ACH.

**Results:**

Among 6 patients (2 males, 4 females; mean age 42.8 years, range 22–59; mean disease duration 1.9 years, range 6 months–4 years), all received upadacitinib 15 mg/day for 3–13 months. Most improved in skin and nail lesions; mean DLQI decreased from 14.0 to 6.0. Response was assessed primarily by clinical signs: complete response required resolution of pustules and erythema with nail improvement; partial response required ≥50% clinical improvement. One patient had clinical complete response (DLQI 71%) and another clinical partial response (DLQI 42%). No adverse events occurred. Notably, 3 patients who failed tofacitinib improved after switching, though this is preliminary and may be confounded by cumulative exposure.

**Conclusion:**

In this small retrospective case series, upadacitinib 15 mg/day appeared effective in ACH, with most patients showing clinical improvement, although two had subthreshold DLQI improvement despite clinical response. The literature review indicates that JAK inhibitors, particularly upadacitinib, show promising efficacy in ACH, but current evidence remains limited to case reports and small series. Response after switching from tofacitinib in three patients is preliminary and may be confounded by cumulative exposure; larger prospective studies are needed to confirm efficacy, safety, and the value of switching.

## 1 Introduction

Acrodermatitis continua of Hallopeau (ACH) is a relatively rare chronic skin disorder that primarily affects the acral regions of the body, such as the fingers and toes. This condition is often associated with immune system abnormalities, leading to symptoms including erythema, scaling, and pruritus, and can even progress to distal phalangeal osteolysis, severely impairing hand function and quality of life (1, 2). ACH is considered a localized subtype of pustular psoriasis and shares histopathological features (subcorneal neutrophilic accumulation) with palmoplantar pustulosis (PPP) and generalized pustular psoriasis (GPP) (3–5). Epidemiological data indicate that ACH accounts for a certain proportion of pustular psoriasis cases and exhibits both phenotypic overlap and differences when compared with PPP (4–6).

Due to the rarity of ACH, there is currently no unified clinical guideline. Conventional therapies include acitretin, cyclosporine, and methotrexate, but they have limited efficacy and significant adverse effects. Yuan et al. (7) analyzed 293 systemic treatment episodes in 96 ACH patients and reported an overall good response rate of only 26.3%, with acitretin achieving a 30% good response rate. Kromer et al. (8) reported 39 ACH patients, most of whom responded poorly to conventional treatments. Regarding biologics, TNF-α inhibitors are widely used, but some patients experience secondary failure (9, 10); IL-17 inhibitors and IL-12/23 inhibitors have also been reported to be effective in individual cases (11–13). A systematic review by Maliyar et al. (10) summarized the use of biologics in ACH, noting that targeted therapies hold promise but the level of evidence remains low. Sun et al. (14) explicitly identified JAK inhibitors as an emerging direction for ACH treatment (15, 16), yet direct evidence is extremely scarce (accounting for only 1.0% of all targeted therapies). Furthermore, spesolimab (an IL-36 receptor inhibitor) has shown efficacy in ACH patients coexisting with GPP (13).

The JAK-STAT pathway is a key node in the signal transduction of multiple pro-inflammatory cytokines, including IL-6, IL-12, IL-17, IL-23, and IFN-γ. Among JAK inhibitors, upadacitinib is a highly selective JAK1 inhibitor. This selectivity is particularly relevant for ACH, as the IL-23/Th17 axis, a central pathogenic pathway in pustular psoriasis, relies predominantly on JAK1-mediated signaling. Additionally, upadacitinib has been approved for related immune-mediated conditions including psoriatic arthritis and atopic dermatitis, with a well-characterized safety profile and rapid onset of action. In contrast, pan-JAK or JAK1/3 inhibitors may have broader but less targeted effects. Upadacitinib is a highly selective JAK1 inhibitor that has been approved for diseases such as atopic dermatitis and psoriatic arthritis, but its use in ACH has only been reported in isolated cases (17–19). Recent real-world evidence supports upadacitinib in other neutrophilic dermatoses such as hidradenitis suppurativa, providing additional rationale for its off-label use in ACH (20). This study reports the efficacy and safety of upadacitinib in six patients with ACH, and, combined with a literature review, explores the potential value of JAK inhibitors in the treatment of ACH as well as the switching strategy.

## 2 Case series

### 2.1 Patients and methods

Six consecutive patients diagnosed with ACH at the dermatology outpatient clinic of the First Affiliated Hospital of Chongqing College of Traditional Chinese Medicine from January 2023 to December 2025 and treated with upadacitinib were enrolled. All patients met the clinical and histopathological diagnostic criteria for ACH. Prior to initiating upadacitinib, all patients underwent a standardized washout period. For patients on acitretin (n=6), the washout period was 2 weeks. For the 3 patients previously treated with tofacitinib, a 1-week washout period was implemented, consistent with the drug’s 3–4 half-lives. Antihistamines were continued during the washout period as needed for symptom control. Routine screening, including complete blood count, liver and kidney function, and other relevant examinations, was performed before treatment, and all results were normal. Upadacitinib was initiated at a dose of 15 mg once daily. Efficacy assessment included clinical improvement of skin lesions (pustules, erythema, nail changes) and changes in the Dermatology Life Quality Index (DLQI) score. Adverse events were recorded through regular follow-up visits.

Treatment response was assessed primarily by clinical signs (pustules, erythema, scaling, and nail involvement) and secondarily by DLQI. Complete response was defined as complete resolution of pustules and erythema with marked improvement or normalization of nail changes; partial response as ≥50% reduction in pustules and/or nail involvement not meeting complete response criteria; and no response as <50% improvement in clinical signs or disease progression. DLQI improvement (≥75% for complete, ≥50% for partial) was recorded as supportive only, as DLQI has not been validated specifically for ACH. Time to initial response was the earliest time point with ≥50% pustule reduction; time to maximal response was the time point of best clinical improvement (pustules, erythema, and nail involvement), with DLQI as a supportive reference.

### 2.2 Clinical characteristics and outcomes

Among the six patients, there were two males and four females, with a mean age of 42.8 years (range 22–59 years) and a mean disease duration of 1.9 years (range 6 months to 4 years). Sites involved: five patients had only finger involvement, and one patient had both finger and toe involvement. Prior treatments: all six patients had received acitretin and antihistamines; three of them (Cases 1–3) had previously been treated with tofacitinib (5 mg twice daily or 10 mg/day) for 3–8 months with suboptimal response, whereas the other three (Cases 4–6) had not received tofacitinib. None of the patients had used biologics.

Representative case (Case 2): A 22-year-old female with a 4-year disease duration presented with recurrent periungual pustules, nail plate thickening, pitting, and partial loss involving two fingers of both hands, accompanied by significant pain. She had previously received acitretin for 3 months with poor response, followed by tofacitinib 5 mg twice daily for 3 months, which achieved only partial improvement followed by relapse. After switching to upadacitinib 15 mg/day, pustules began to resolve at 8 weeks, and at 13 months the nail plates had fully regenerated with a normal appearance; the DLQI score decreased to 5, and no adverse events occurred (**Figure 1**). The outcomes of the remaining five patients are summarized in **Table 1**. Most patients responded within 4–8 weeks, with mean DLQI decreasing from 14.0 to 6.0. No adverse events occurred in this small series. According to the predefined criteria, Case 2 was classified as clinical complete response (DLQI 71%, below the supportive threshold), and Case 6 as clinical partial response (≥50% clinical improvement but DLQI 42%, insufficient support).

**Figure 1:**
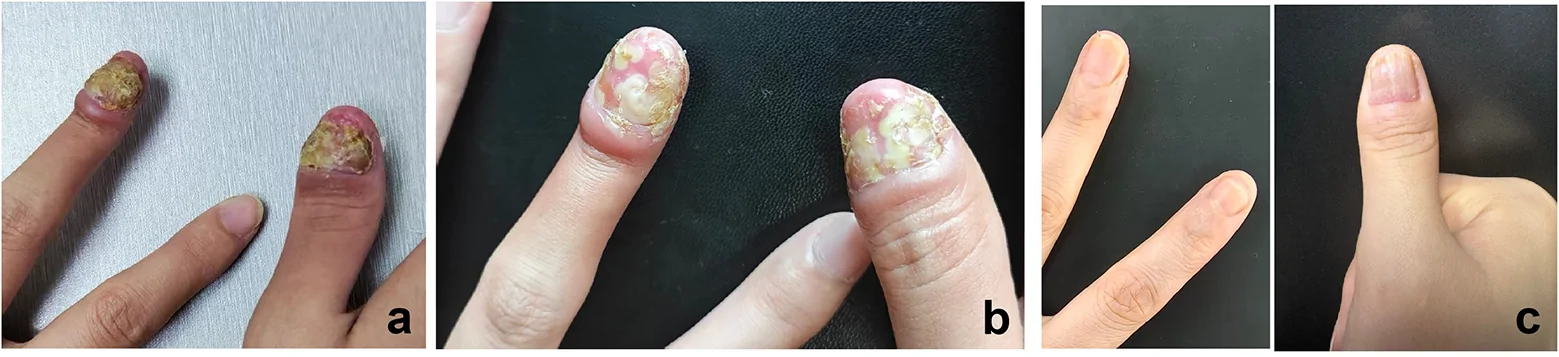
Clinical improvement in Case 2 after upadacitinib treatment. **(a)** Before treatment: marked periungual erythema, swelling, pustules, severe nail plate dystrophy with thickening, pitting, and partial loss of fingernails. **(b)** Reduction of pustules and erosions after 8 weeks of treatment. **(c)** After 13 months of upadacitinib 15 mg once daily: complete resolution of erythema and pustules, significant nail regrowth with smooth and healthy appearance.

**Table 1:** Clinical data and outcomes of 6 ACH patients.

Case	Age/sex	Disease duration	Sites involved	Prior therapies	Treatment duration	Time to initial response (weeks)	Time to maximal response (weeks)	DLQI (baseline → endpoint)	DLQI assessment time point	Response category	Sustained remission	Adverse events
1	34/F	2 years	Fingers	AHs, AC, tofacitinib	6 months	4	12	14 → 6	Month 6	Partial (57%)	Yes	None
2	22/F	4 years	Fingers	AHs, AC, tofacitinib	13 months	4	8	17 → 5	Month 13	Compelte(71%,(below supportive threshold))	Yes	None
3	59/M	3 years	Fingers, toes	AHs, AC, tofacitinib	8 months	6	12	16 → 6	Month 8	Partial (63%)	Yes	None
4	52/F	6 months	Fingers	AHs, AC	3 months	4	8	12 → 6	Month 3	Partial (50%)	Yes	None
5	50/M	8 months	Fingers	AHs, AC	5 months	6	12	13 → 6	Month 5	Partial (54%)	Yes	None
6	40/F	1 year	Fingers	AHs, AC	5 months	8	12	12 → 7	Month 5	Partial (42%,insufficient support)	Yes	None

F, female; M, male; AHs, antihistamines; AC, acitretin capsules; DLQI, Dermatology Life Quality Index. “Time to initial response” defined as the earliest time point with ≥50% pustule reduction. “Time to maximal response” defined as the time point achieving the best clinical improvement (based on pustules, erythema, and nail involvement), with DLQI used as a supportive reference. Clinical response categories: Complete response = complete resolution of pustules and erythema with marked improvement or normalization of nail changes; Partial response = ≥50% reduction in pustules and/or nail involvement but not meeting complete response criteria; No response = <50% improvement in clinical signs or disease progression. DLQI improvement was recorded as a supportive measure (≥75% for complete response, ≥50% for partial response). “Sustained remission” indicates no relapse during the follow-up period. No adverse events were observed in this small series.

## 3 Literature review

### 3.1 Current treatment landscape of ACH

Due to its extremely low incidence, the treatment of ACH lacks standardized protocols. Yuan et al. (7) reviewed 96 patients and reported an overall good response rate of only 26.3% for systemic therapy. Although acitretin is the first-line preferred agent, its good response rate is only 30.0%, and relapse after discontinuation is common. Kromer et al. (8) reported 39 patients with ACH, most of whom responded poorly to conventional treatments. An analysis of 65 ACH patients by Chularojanamontri et al. (21) further confirmed the limited efficacy of conventional therapies. Regarding biologics, TNF-α inhibitors (e.g., adalimumab) are the most widely used, but some patients experience secondary failure (9, 10); IL-17 inhibitors (e.g., secukinumab, ixekizumab, bimekizumab, vunakizumab) and IL-12/23 inhibitors (e.g., ustekinumab) have also been reported to be effective in individual cases (11, 12, 22). A systematic review by Maliyar et al. (10) summarized the use of biologics in ACH, noting that targeted therapies hold promise but the level of evidence remains low. A clinical perspectives article by Smith et al. (23) also emphasized the challenges in managing ACH. Furthermore, spesolimab (an IL-36 receptor inhibitor) has shown efficacy in ACH patients with coexisting GPP (13).

A systematic review by Sun et al. (14) indicated that JAK inhibitors are an emerging direction for ACH treatment, but direct evidence remains extremely scarce (accounting for only 1.0% of all targeted therapies). In the clinical management algorithm for ACH published in JAAD, Iorizzo et al. (24) positioned acitretin as first-line and biologics and small-molecule targeted agents as second-/third-line options, while also noting that “currently no drug can achieve and maintain long-term remission.”

### 3.2 Direct evidence of JAK inhibitors in ACH

We searched PubMed, Web of Science, and Embase for original articles on JAK inhibitors for the treatment of ACH published up to May 2026, and identified several core case reports (**Table 2**). The drugs covered tofacitinib, upadacitinib, and baricitinib, used either as monotherapy or in combination. All cases showed clinical improvement and no serious adverse events.

**Table 2:** Summary of published case reports of JAK inhibitor therapy for acrodermatitis continua of Hallopeau.

Author, year	Age/sex	Disease duration	Sites involved	Prior systemic therapies	JAK inhibitor (target), dosage	Treatment duration	Time to response	Outcome	Adverse events
Shen D et al., 2024 (19)	47/F	1 year	Left 3rd and 5th fingers	Topical calcipotriol betamethasone (limited); declined acitretin, cyclosporine, methotrexate	Tofacitinib (JAK1/3), 5 mg bid	12 weeks	4 weeks (gradual); 12 weeks (complete)	Complete clearance; DLQI 8→1	None
Yang N et al., 2025 (26)	52/F	10 years	Fingertips; flexural areas (groin, perianal, axilla)	Acitretin, apremilast; ixekizumab, adalimumab, secukinumab, guselkumab (all failed for ACH)	Upadacitinib (JAK1), 15 mg/d → 15 mg every 3 days	9 months	1 week (rapid); 2 weeks (significant); 4 weeks (near-complete)	Complete remission of skin and nails; joint symptoms controlled	None
Long F et al., 2026 (17)	51/F	1 year	Multiple fingertips (5 nails with complete loss)	Topical antifungals, corticosteroids (misdiagnosed)	Upadacitinib (JAK1), 15 mg/d	1 year (ongoing)	12 days	Complete remission; nail regrowth	None
Muto Y et al., 2025 (18)	70/M	Refractory (NS)	Distal digits (fingers)	Topical corticosteroids, vitamin D3, oral antibiotics; apremilast (temporary, then recurrence), etretinate (discontinued), deucravacitinib (no improvement)	Baricitinib (JAK1/2), 4 mg/d	≥4 weeks	4 weeks	Marked improvement; no recurrence	None
Han GM et al., 2021 (16)	28/F	Progressive (NS)	Fingernails with distal interphalangeal joint swelling (both hands)	Oral cefuroxime; retinoids (30 mg/d) + topical corticosteroids ×12 weeks; methotrexate (15 mg/week) + topical corticosteroids ×12 weeks (all failed)	Baricitinib (JAK1/2), 2 mg/d	5 months	5 days	Clinical remission (pustules resolved, joint swelling improved); no new bone erosions	None
Chen X et al., 2023 (15)	31/F	9 months	Palms, soles; scalp (psoriasiform lesions), alopecia	Ixekizumab (160 mg → 80 mg q2w ×2 months, no improvement); infliximab (5 mg/kg, paradoxical reaction after 3rd dose)	Ustekinumab (45 mg) + Tofacitinib (JAK1/3, 5 mg bid)	8 months	NS	Lesions almost disappeared; nearly all nails recovered normal; flare after dose reduction	Not reported

Chen et al. (2023) used combination therapy (ustekinumab + tofacitinib); therefore, it is not a JAK inhibitor monotherapy.”NS” = not specified in the original publication. All reported cases showed clinical improvement with no serious adverse events.

The above direct evidence indicates that JAK inhibitors, especially the highly selective JAK1 inhibitor upadacitinib, can rapidly and persistently control ACH activity and are well tolerated. Notably, in the present case series, three patients who had previously failed to respond to tofacitinib showed clinical improvement after switching to upadacitinib, suggesting the possibility of benefit from switching between different JAK inhibitors.

### 3.3 Indirect evidence: JAK inhibitors in PPP and inflammatory nail disorders

ACH, PPP, and GPP belong to the group of neutrophilic pustular dermatoses and share the IL-23/IL-17 axis pathogenic mechanism. Iorizzo et al. (25) systematically reviewed the clinical outcomes of JAK inhibitors in inflammatory nail disorders (including PPP), showing that JAK inhibitors can significantly improve nail psoriasis and pustular nail disease. Although no large-scale prospective trials are available, several case series have confirmed that upadacitinib, tofacitinib, and other JAK inhibitors are effective in PPP, with rapid onset and good safety. These indirect findings further support the rationale for using upadacitinib in the treatment of ACH.

## 4 Discussion

### 4.1 Efficacy analysis of upadacitinib in the treatment of ACH

Most patients in this case series achieved significant clinical improvement after treatment with upadacitinib 15 mg/day, with an onset of response within 4–8 weeks and a mean DLQI reduction of 57%, consistent with the efficacy of JAK inhibitors in ACH reported in the literature (17, 19, 26). Three patients who had previously failed tofacitinib showed improvement after switching to upadacitinib. However, with only three patients, we cannot exclude cumulative exposure to prior JAK inhibition or delayed effects. This observation should be considered hypothesis-generating rather than definitive evidence for a switching strategy.

### 4.2 Clinical implications of a JAK inhibitor switching strategy

In this series, three patients who failed tofacitinib responded to upadacitinib. This observation raises the possibility of a JAK inhibitor switching strategy in ACH, which has not been previously reported. The mechanistic rationale lies in the differential kinase selectivity of these agents. The IL-23/Th17 axis, central to ACH, signals through JAK1, which mediates IL-6, IL-12, and IL-23 signaling in ACH. Tofacitinib also inhibits JAK3, which signals through IL-2, IL-4, IL-15, and IL-21, pathways less relevant to ACH. Upadacitinib’s JAK1 selectivity offers more targeted inhibition. Evidence from related pustular disorders supports this approach. PPP shares this pathophysiology with ACH, and JAK inhibitors including tofacitinib and upadacitinib are effective in PPP (27–29). Though switching studies are lacking, overlapping cytokine networks suggest that differential JAK selectivity yields different outcomes. Failure of a less selective JAK inhibitor does not predict failure of a more selective one when the dominant pathway is JAK1-dependent. JAK inhibitors also show efficacy in psoriatic arthritis (30). These findings suggest a possible benefit of switching from tofacitinib to upadacitinib; however, this is a preliminary observation from three patients and requires prospective validation. Future biomarker studies, including cytokine profiling and IL36RN genotyping, may identify optimal candidates and timing for switching.

### 4.3 Comparison with other targeted therapies

In addition to JAK inhibitors, various biologic agents have also shown efficacy in ACH. Adalimumab (a TNF-α inhibitor) has successfully treated several ACH patients (9, 10); IL-17 inhibitors, such as secukinumab, ixekizumab, bimekizumab, and vunakizumab, have been reported to be effective in individual cases (11, 12, 22); the IL-23 inhibitor guselkumab has also been used (26); and spesolimab (an IL-36 receptor inhibitor) has shown efficacy in ACH coexisting with GPP (13). However, most of these biologics target a single cytokine, whereas JAK inhibitors can block multiple downstream inflammatory pathways simultaneously, theoretically offering a broader spectrum of anti-inflammatory effects. In this study, upadacitinib showed clinical improvement in patients who had failed tofacitinib, further reflecting the differences in target coverage among different agents.

### 4.4 Potential for long-term remission

In the ACH clinical management algorithm published in JAAD, Iorizzo et al. (24) noted that despite the availability of multiple targeted treatments, currently no drug can achieve and maintain long-term remission. However, Han et al. (31) challenged this view and reported a case of sustained remission for 3 years and 7 months after discontinuation of baricitinib. In the present series, Case 2 maintained clinical complete response under continuous treatment for 13 months, and Cases 1–3 remained effective after switching from tofacitinib to upadacitinib, further suggesting that with appropriate selection of JAK inhibitors, some ACH patients may achieve long-term disease control or even remission after drug withdrawal.

### 4.5 Safety

No adverse events occurred during 3–13 months of follow-up; however, the small sample size precludes definitive safety conclusions. Although larger prospective studies are needed, our experience is consistent with real-world data for upadacitinib in hidradenitis suppurativa (20). Routine monitoring of blood counts, liver and kidney function, and lipids is advised (17–19, 26).

### 4.6 Limitations and future directions

This study has several limitations. It is a retrospective case series with a small sample size (n=6) and no control group. Standardized clinical photographs were not systematically available for all patients; only Case 2 had complete photographic documentation. To mitigate this, we supplemented our assessment with quantitative DLQI trajectories (**Figure 2**) and predefined response categories (**Table 1**). We also lacked a validated ACH-specific severity scoring tool, relying instead on descriptive clinical assessment with DLQI as a supportive measure. The longest follow-up was 13 months, precluding assessment of long-term safety and durability of response after drug withdrawal. Prospective studies with standardized photographic documentation and validated severity scores are warranted.

**Figure 2:**
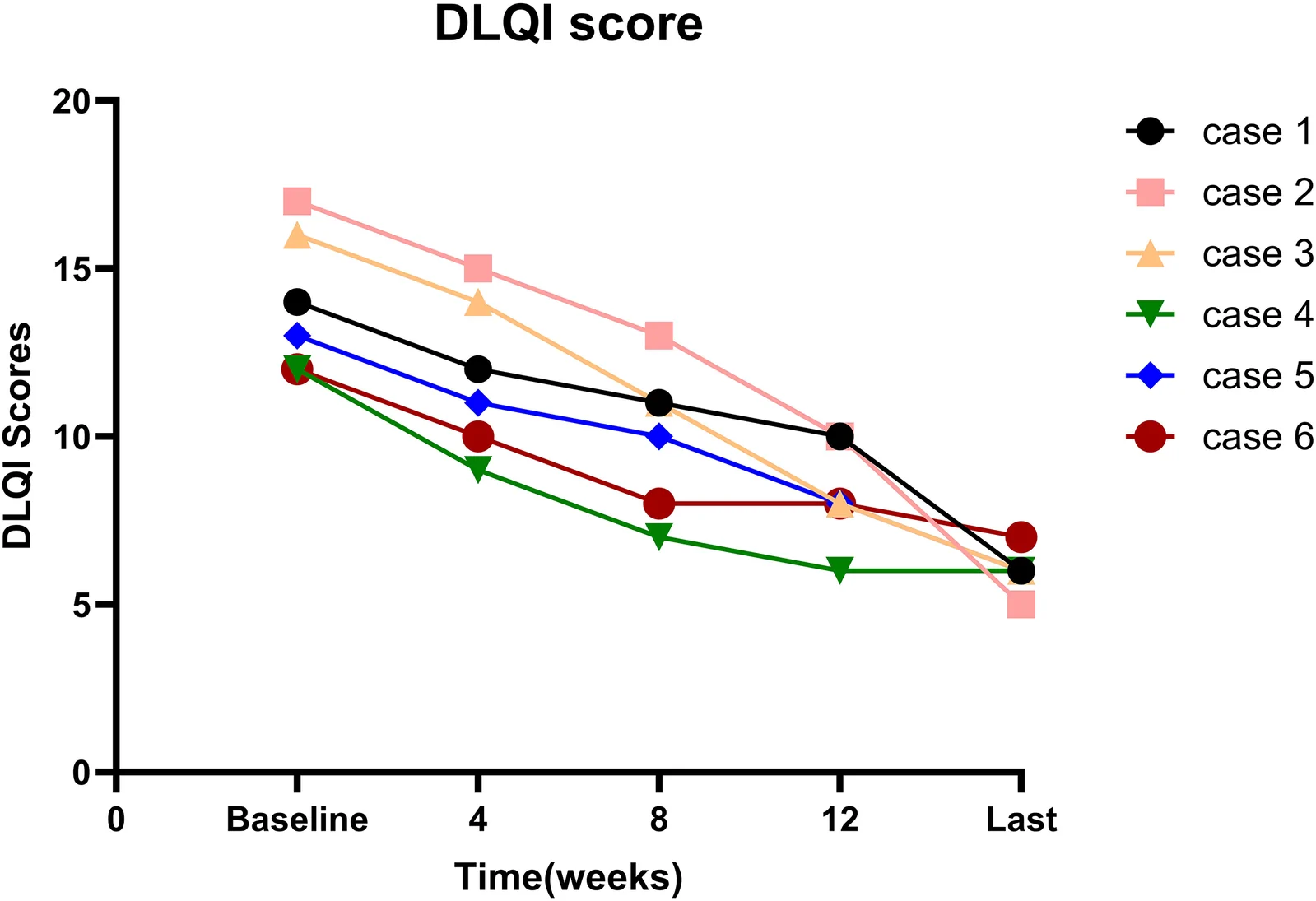
DLQI trajectory over time in six ACH patients treated with upadacitinib 15 mg/day. Individual DLQI scores at baseline, week 4, week 8, week 12, and at the final follow-up visit for all six patients. All six patients showed DLQI improvement, but two had subthreshold improvement relative to the predefined supportive thresholds (≥75% for complete response, ≥50% for partial response).

## 5 Conclusion

In this small retrospective case series, upadacitinib 15 mg/day appeared effective in ACH, with most patients showing clinical improvement; however, two patients had subthreshold DLQI improvement despite clinical response. The observation that three patients responded after switching from tofacitinib is preliminary and may be confounded by cumulative exposure. Larger prospective studies are needed to confirm efficacy, safety, and the value of switching.

## Data Availability

The original contributions presented in the study are included in the article/supplementary material, further inquiries can be directed to the corresponding author/s.
